# Supramolecular Nanoparticles of Histone and Hyaluronic Acid for Co-Delivery of siRNA and Photosensitizer In Vitro

**DOI:** 10.3390/ijms25105424

**Published:** 2024-05-16

**Authors:** Minxing Hu, Jianwei Bao, Yuanmei Zhang, Lele Wang, Ya Zhang, Jiaxin Zhang, Jihui Tang, Qianli Zou

**Affiliations:** 1 Research and Industrialization of New Drug Release Technology Joint Laboratory of Anhui Province, School of Pharmacy, Anhui Medical University, Hefei 230032, China; 2 Institute of Health and Medicine, Hefei Comprehensive National Science Center, Hefei 230000, China

**Keywords:** histone, hyaluronic acid, photodynamic therapy, siRNA, supramolecular assembly

## Abstract

Small interfering RNA (siRNA) has significant potential as a treatment for cancer by targeting specific genes or molecular pathways involved in cancer development and progression. The addition of siRNA to other therapeutic strategies, like photodynamic therapy (PDT), can enhance the anticancer effects, providing synergistic benefits. Nevertheless, the effective delivery of siRNA into target cells remains an obstacle in cancer therapy. Herein, supramolecular nanoparticles were fabricated via the co-assembly of natural histone and hyaluronic acid for the co-delivery of HMGB1-siRNA and the photosensitizer chlorin e6 (Ce6) into the MCF-7 cell. The produced siRNA-Ce6 nanoparticles (siRNA-Ce6 NPs) have a spherical morphology and exhibit uniform distribution. In vitro experiments demonstrate that the siRNA-Ce6 NPs display good biocompatibility, enhanced cellular uptake, and improved cytotoxicity. These outcomes indicate that the nanoparticles constructed by the co-assembly of histone and hyaluronic acid hold enormous promise as a means of siRNA and photosensitizer co-delivery towards synergetic therapy.

## 1. Introduction

Cancer is a complex and devastating disease that poses a significant threat to global health [1]. Currently available cancer therapies, including surgery, chemotherapy, and radiation therapy, have indeed demonstrated notable progress in advancing patient outcomes [2,3]. However, these therapeutic interventions frequently produce severe side effects and lack precision by damaging both healthy and cancerous cells [4]. To overcome these limitations, it is imperative to devise innovative and targeted therapeutic strategies. A promising approach is the use of small interfering RNA (siRNA) as a potential therapy, which has received widespread recognition. siRNA is a potent tool, capable of selectively silencing genes that are implicated in the progression of cancer. By utilizing the natural mechanism of RNA interference, siRNA-based therapies display substantial potential for addressing the molecular drivers of cancer [5]. However, a significant hurdle exists in delivering siRNA molecules to the cancer location since they are chemically unstable and rapidly degraded by nucleases present in the bloodstream. Additionally, effectively and directly delivering siRNA to cancer cells while avoiding off-target effects on normal cells remains a substantial challenge [6,7]. Various types of nanocarriers, such as liposomes [8,9], polymer nanoparticles [10,11], metal nanoparticles [12,13], and peptide nanoparticles [14,15], have been investigated for delivering siRNA. However, only a limited number of them, such as cationic liposomes, have advanced to clinical applications [16]. Therefore, additional research is warranted to explore efficient delivery systems.

The supramolecular co-assembly of biomolecules occurs frequently in living organisms [17]. The interaction between two biomolecules carrying opposite charges is predominantly based on electrostatic interactions. These interactions can be utilized as the foundation for the fabrication of nanomaterials using the co-assembly approach. Co-assembled nanomaterials have been demonstrated using various charged biomolecules, such as proteins, polypeptides, and hyaluronic acid (HA), as self-assembling building blocks and investigated for the delivery of therapeutic agents, such as photosensitizers for photodynamic therapy (PDT) [18], chemotherapeutic drugs for chemotherapy [19], and heparin for anti-thrombus therapy [20]. Especially nanomaterials consisting of two oppositely charged biomolecules have shown the ability to load multiple molecules with distinct properties [21], providing the possibility to realize the co-delivery of two therapeutic agents for synergetic therapy. The synergetic therapy based on the co-delivery of drugs with different therapeutic mechanisms, such as PDT and chemotherapy, has produced significant benefits when compared with single treatments [22]. Histone is a widely acknowledged positively charged protein and structural supporter for chromosomes, operating through electrostatic interactions with DNA. Consequently, histone has undergone extensive biomolecule investigation for DNA binding [23,24]. However, the application of histone as a building block in a co-assembly system toward the co-delivery of nucleic acid and other therapeutic agents has yet to be demonstrated.

**Scheme 1:**
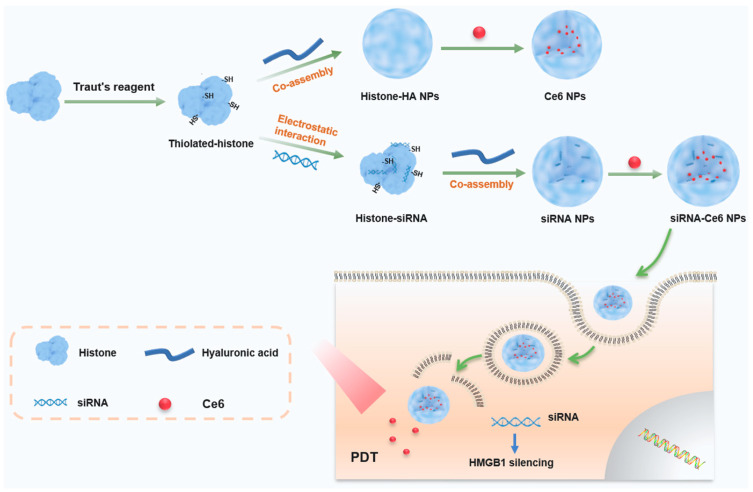
Illustration of histone-and-HA-based nanoparticles as photosensitizers and siRNA delivery nanocarriers for combined therapy of breast cancer.

Herein, we fabricated supramolecular nanoparticles through a co-assembly approach using positively charged histone and negatively charged HA as the building blocks (Scheme 1). siRNA that targets HMGB1 and chlorin e6 (Ce6), a widely investigated photosensitizer for PDT, were facilely incorporated in the histone-HA nanoparticles (histone-HA NPs), generating siRNA-Ce6-loaded nanoparticles (siRNA-Ce6 NPs). The in vitro results demonstrated that the siRNA-Ce6 NPs combined gene therapy and PDT and thus enhanced the cytotoxic effects on cancer cells. The exploration of histone as the charged building block in co-assembly is expected to promote the development of multifunctional drug delivery systems, particularly in the applications of novel anticancer treatments such as gene therapy and PDT.

## 2. Results and Discussion

### 2.1. Assembly and Characterization of Histone-HA NPs and siRNA-Ce6 NPs

**Figure 1:**
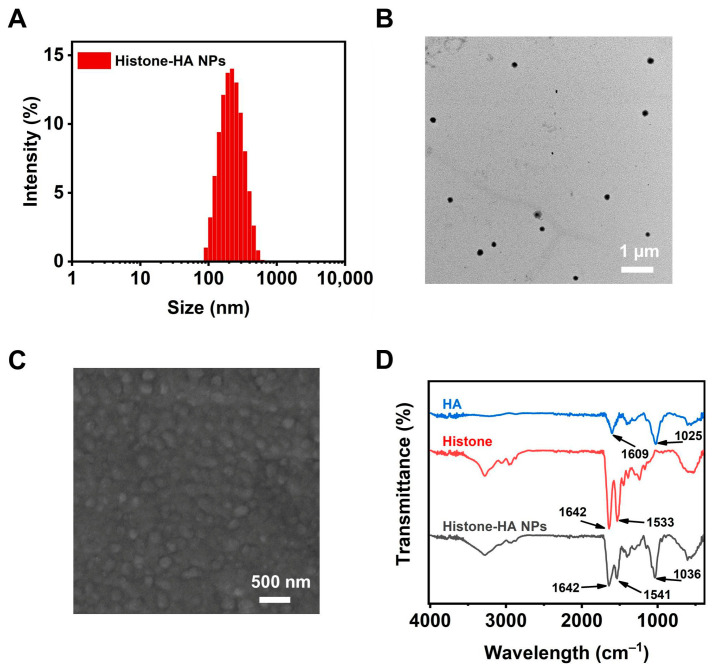
Characterization of histone-HA NPs. (**A**) Dynamic light scattering (DLS) measurement results. (**B**) TEM images of histone–HA NPs. (**C**) SEM images of histone-HA NPs. (**D**) FTIR spectra of histone-HA NP powders.

The histone-HA NPs were formed through the co-assembly of thiolated histone and HA in aqueous solution driven by electrostatic interactions. The thiolation was realized by using Traut’s reagent to introduce thiol groups onto the protein surface. The subsequent crosslinking resulted in the formation of stable histone-HA NPs. The chemical modification by thiol groups and further co-assembly with HA could alter the natural structure of histone, providing the possibility to overcome or reduce the immunogenicity of histone [25]. As shown in Figure 1A, the mean hydrodynamic diameter of the histone-HA NPs was found at 195.4 nm measured by dynamic light scattering (DLS). The polydispersity index value was determined to be 0.170, indicating a narrow particle size distribution. Particularly, the average size of the histone-HA NPs exhibited no discernible change over a 7-day aging period (Figure S1), suggesting that these nanoparticles possess excellent structural stability in solution. In addition, the surface charge of histone-HA NPs was found to be negative with a zeta potential at −28.2 mV. The scanning electron microscopy (SEM) and transmission electron microscopy (TEM) images indicated that histone-HA NPs are uniform spheres, which is almost in agreement with diameters measured by DLS (Figure 1B,C). Nanoparticles can passively accumulate in cancers more than in normal tissues due to the enhanced permeability and retention (EPR) effect [26]. Furthermore, negatively charged nanoparticles may have a longer circulation time [27,28]. Therefore, the properties of histone-HA NPs indicated that they are promising drug delivery carriers for therapeutic agents. Histone-HA NPs were further characterized by using Fourier transform infrared (FTIR) spectroscopy (Figure 1D). In histone-HA NPs, the stretching vibration of the C-O of HA shifted from 1025 to 1036 cm^−1^, and the stretching vibration of the C=O of HA at 1609 cm^−1^ was replaced by histone at 1642 cm^−1^. The N-H scissoring vibration of histone shifted from 1533 to 1541 cm^−1^, indicating the presence of electronic interaction in histone-HA NPs.

To evaluate the drug loading performance of histone-HA nanoparticles, photosensitizer Ce6 and HMGB1-siRNA were used as model drugs. Ce6 is a typical second generation of photosensitizers used in PDT with low dark toxicity and efficient reactive oxygen species (ROS) production under laser irradiation. ROS-induced cytotoxicity can selectively destroy cancer cells while minimizing damage to healthy tissues [29]. HMGB1 has been found to exert diverse effects that contribute to tumor growth, metastasis, and resistance to therapy [30,31]. The multifaceted actions of HMGB1 highlight its crucial role in shaping the tumor microenvironment and influencing cancer progression [32,33]. As such, targeting HMGB1 holds potential for developing novel therapeutic strategies to combat cancer.

**Figure 2:**
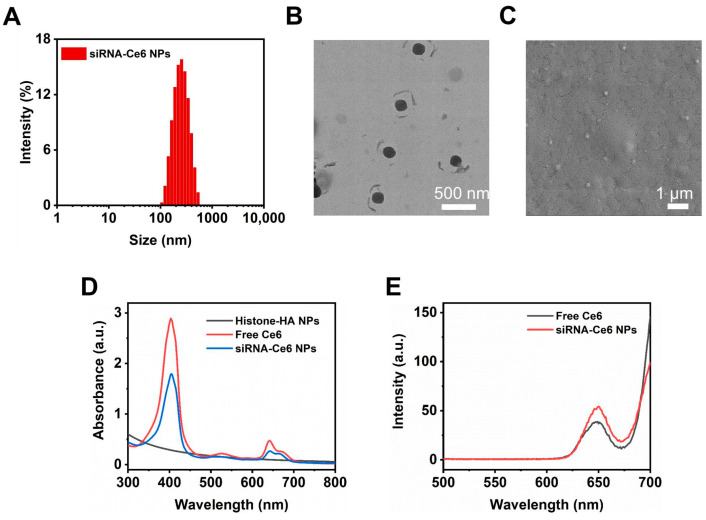
Characterization of siRNA-Ce6 NPs. (**A**) Dynamic light scattering (DLS) measurement results. (**B**) TEM images of siRNA-Ce6 NPs. (**C**) SEM images of siRNA-Ce6 NPs. (**D**) UV-Vis spectra of siRNA-Ce6 NPs. (**E**) Fluorescence spectrum of siRNA-Ce6 NPs.

For the preparation of siRNA-Ce6 NPs, a complex of siRNA and histone was prepared through electrostatic interaction, which was then combined with HA and Ce6 to form nanoparticles during the self-assembly process. The average diameter of the siRNA-Ce6 NPs was measured by DLS and found to be 226.3 nm, with a PDI of 0.142 (Figure 2A). Importantly, siRNA-Ce6 NPs also possess excellent structural stability over a 7-day aging period (Figure S2). Compared to the blank carriers, the particle size slightly increased after drug loading. There was no significant change in zeta potential, and it was measured to be −27.7 mV. SEM and TEM showed that siRNA-Ce6 NPs maintain the spherical morphology (Figure 2B,C). In addition, the UV–visible absorption spectrum of the siRNA-Ce6 NP solution reveals distinct absorption peaks of Ce6 in the UV spectrum at 405 nm and 664 nm (Figure 2D). Furthermore, the fluorescence spectrum shows a fluorescence signal of Ce6 at 650 nm (Figure 2E). The optical properties of siRNA-Ce6 NPs demonstrated a successful incorporation of Ce6 into the nanoparticles. The encapsulation efficiency and drug loading capacity of Ce6 in the siRNA-Ce6 NPs were determined to be 72.43 ± 1.44% and 3.49 ± 0.07%, respectively. For siRNA, the encapsulation efficiency was found to be 87.48 ± 0.34%. The loading of siRNA is primarily due to electrostatic interactions, where the negative charge of siRNA is attracted to the positive charge of histone, while the loading of Ce6 is predominantly driven by hydrophobic effects, given that Ce6 is a hydrophobic molecule. These findings support the potential of the developed siRNA-Ce6 nanoparticles as an effective therapeutic strategy for future applications.

### 2.2. Intracellular Uptake and Cytotoxicity of siRNA-Ce6 NPs

**Figure 3:**
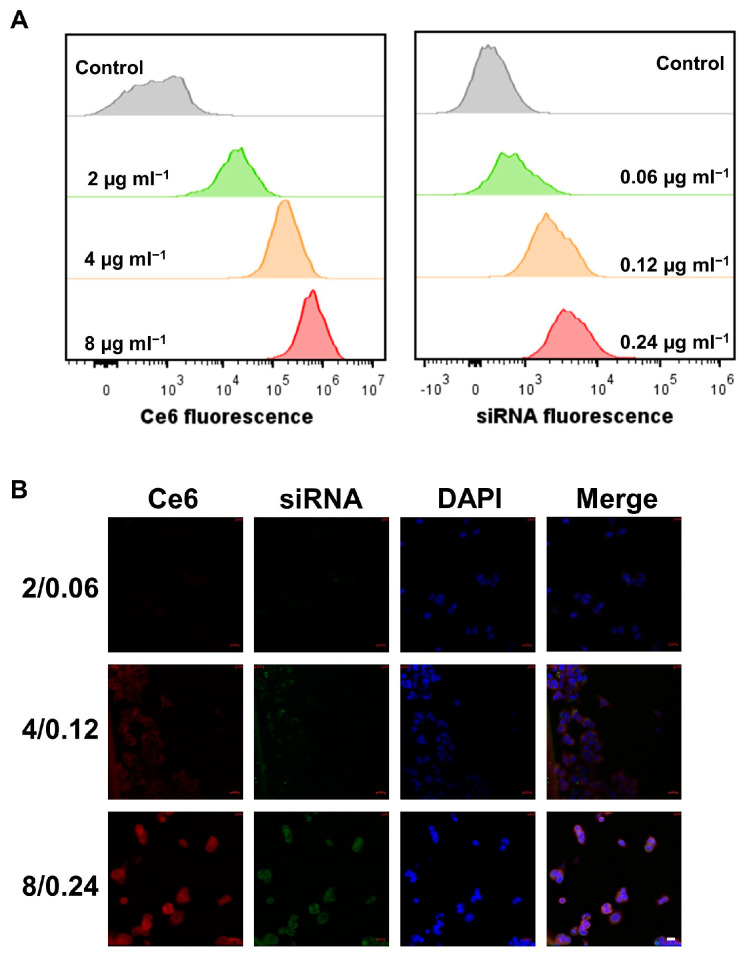
Cellular uptake of siRNA-Ce6 NPs. (**A**) Flow cytometry analysis and (**B**) CLSM images of siRNA-Ce6 NPs incubated with MCF-7 cells for 24 h. Concentrations of Ce6 in nanoparticles were 2, 4, and 8 μg mL^−1^, respectively, and siRNA were 0.06, 0.12, and 0.24 μg mL^−1^, respectively. Scale bar: 20 μm.

Intracellular Ce6 and siRNA delivery in MCF-7 cells was evaluated through flow cytometry and confocal microscopy (CLSM) (Figure 3A,B). After 24 h of incubation with the siRNA-Ce6 NPs, the cellular levels of Ce6 and siRNA increased with higher nanoparticle concentrations. However, when the cells were incubated with the nanoparticles for only 2 h, limited cellular uptake was observed (Figure S3). This observation indicates that the nanoparticles effectively deliver Ce6 and siRNA into the cells in a concentration- and time-dependent manner.

**Figure 4:**
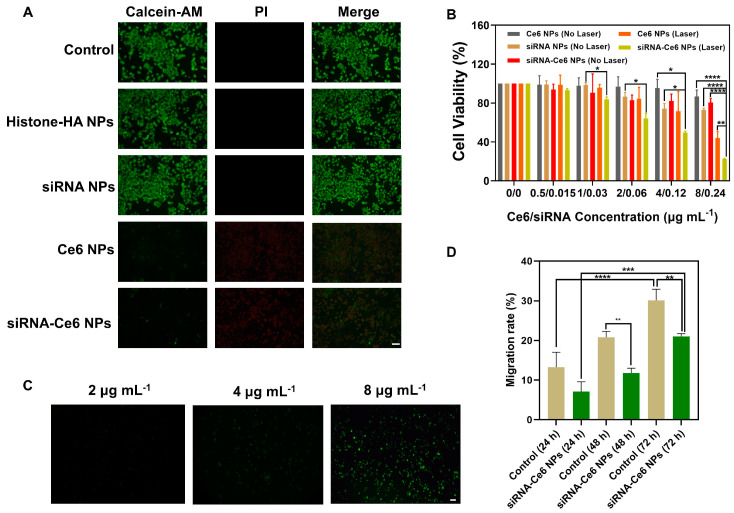
(**A**) Calcein-AM and PI live–dead cell staining of MCF-7 cells after treatment with histone–HA NPs, siRNA NPs, Ce6 NPs, and siRNA-Ce6 NPs with laser irradiation (660 nm, 0.2 W cm^−2^, 1 min). Living cells were stained green with calcein-AM, and dead cells were stained red with PI. (**B**) Cell viabilities of MCF-7 cells after treatment with different formulations for 24 h and subjected to laser irradiation. (**C**) Fluorescence imaging of MCF-7 cells stained with DCFH-DA upon 1 min light irradiation after a 24 h incubation with siRNA-Ce6 NPs (2, 4, 8 μg mL^−1^ Ce6). (**D**) Wound healing assay. Analysis of migration rate of MCF-7 cells after treatment with siRNA-Ce6 NPs for 24 h, 48 h, and 72 h. Data are presented as mean ± SEM of three independent experiments. * *p* < 0.05, ** *p* < 0.01, *** *p* < 0.001, **** *p* < 0.0001. Scale bar: 50 μm.

The cytotoxicity of the delivery carriers on cells is an important consideration in evaluating their suitability for therapeutic applications. MTT assays showed that histone–HA NPs lacked significant toxicity to MCF cells even at a high carrier concentration of 1000 μg mL^−1^, suggesting an excellent biocompatibility of carriers (Figure S4). Subsequently, we further evaluated the photoactivity of siRNA-Ce6 NPs in MCF-7 cells, and the results are shown in Figure 4B. Comparing the treatment of cells with Ce6 NPs and Ce6-siRNA NPs alone, both showed significant cellular toxicity with increasing concentration after 1 min of irradiation with a 660 nm laser at an intensity of 0.2 W cm^−2^, indicating the effective PDT of Ce6. Moreover, compared to single treatments of PDT or gene therapy alone, the combination of PDT and gene therapy demonstrates a higher level of cell inhibitory effects. Particularly, the combination index values were smaller than 1 under all the evaluated levels (Figure S5), suggesting that the combination of gene therapy and PDT is synergistic [34]. After staining with the LIVE/DEAD assay kit, significant cellular toxicity induced by PDT can also be easily observed using CLSM. Live cells and dead cells can be easily distinguished by their green and red fluorescence, respectively. As shown in Figure 4A, Ce6 NPs and Ce6-siRNA NPs all killed cells after laser irradiation. However, Ce6 NPs and Ce6-siRNA NPs combined without laser irradiation showed no obvious death (Figure S6).

### 2.3. Investigation on ROS Production and Cell Migration In Vitro

PDT works by using a photosensitizer, light, and oxygen to create reactive oxygen species (ROS), which are highly reactive and can directly damage important structures within cancer cells, such as the membrane and organelles, leading to oxidative stress and subsequent cancer cell death [35,36,37]. Therefore, we evaluated the PDT efficacy of siRNA-Ce6 NPs by measuring intracellular ROS production using the fluorescent probe DCFH-DA. The confocal images of cells treated with various treatments are shown in Figure 4C. The cells treated with siRNA-Ce6 nanoparticles followed by laser irradiation showed an enhanced green fluorescence with increasing concentration, suggesting high intracellular ROS levels and the strong PDT efficacies of Ce6 delivered in the formulation of siRNA-Ce6 NPs.

The migration and invasion of malignant cells play a major role in driving cancer metastasis and recurrence, which became a challenge in cancer treatment [38]. Several studies suggested that targeting HMGB1 through gene therapy can effectively suppress the migration and invasion abilities of breast cancer cells [39,40,41]. Herein, we compared the migration and invasion abilities of MCF-7 cells before and after treatment with siRNA-Ce6 NPs at different time points. As shown in Figure 4D, the migration rate of cells treated with siRNA-Ce6 NPs was significantly lower than that of untreated cells at each time point, indicating that the migration ability of cells was greatly inhibited after silencing HMGB1.

## 3. Materials and Methods

### 3.1. Materials

Histone was purchased from Yuanye Bio-tech (Shanghai, China). HA (MW: 100 KD) was obtained from Heowns Biochem (Tianjin, China). Traut’s reagent was supplied by Sigma-Aldrich (Saint Louis, MO, USA). Ce6 was purchased from Frontier Scientific (Logan, UT, USA). HMGB1-siRNA (sense: *GCUGAAAAGAGCAAGAAAAUU*; antisense: *UUUUCUUGCUCUUUUCAGCCU*) and FAM-labeled HMGB1-siRNA were synthesized by Sangon Biotech (Shanghai, China). Minimum essential medium (MEM), penicillin–streptomycin, fetal bovine serum (FBS), and phosphate buffered solution (PBS) were purchased from Biological Industries (Kibbutz Beit Haemek, Israel). DAPI and thiazolyl blue tetrazolium bromide (MTT) were obtained from Solarbio Sci & Tech (Beijing, China). 2,7-dichlorodihydrofluorescein diacetate (DCFH-DA) was purchased from MedChemExpress (Shanghai, China). The Live/Dead Cell Staining Kit was obtained by Bestbio Biotech (Nanjing, China). The human breast cancer cell line MCF-7 was provided by Procell Life Technology (Wuhan, China).

### 3.2. Preparation of Histone–HA NPs and siRNA-Ce6 NPs

Histone and HA were dissolved separately in ultrapure water at a concentration of 1 mg mL^−1^ each. A total of 10 μL of Traut’s reagent solution (1 mg mL^−1^) was added to the histone solution, and the reaction was carried out at room temperature for 2 h. Afterward, the histone solution and HA solution were mixed in equal volumes and vortexed to form histone–HA nanoparticles. The preparation of siRNA-Ce6 NPs followed a similar process to that of histone–HA NPs. After two hours of the reaction between histone and Traut’s reagent, the siRNA solution (20 μM, 10 μL) was added and incubated for 30 min. Then, the HA solution in the same volume as the histone solution was added and mixed well, followed by a 10 min incubation at room temperature. Finally, the Ce6 solution (10 mg mL^−1^, 10 μL) was added, mixed, and left at room temperature for 30 min to form siRNA-Ce6 nanoparticles. The nanoparticles were characterized and subjected to subsequent experiments after one day of aging.

### 3.3. Characterization of Nanoparticles

The average size and zeta potential of histone–HA NPs and siRNA-Ce6 NPs were determined using a DLS instrument (Malvern Instruments, Malvern, UK). Images of the nanoparticles’ morphology were captured via using scanning electron microscopy (SEM, ZEISS Gemini SEM 300, Oberkochen, Germany) and transmission electron microscopy (TEM, Thermo Scientific Talos L120C G2, Waltham, MA, USA). A drop of the sample was carefully placed onto a 400-mesh carbon-supported copper grid and dried in vacuum, then observed under TEM at 120 kV. UV-Vis absorption spectra and the fluorescence spectrum were recorded using a Shimadzu UV-1900 spectrophotometer (Shimadzu, Tokyo, Japan) and Shimadzu RF-6000 spectrophotometer (Shimadzu, Tokyo, Japan), respectively. FTIR spectra were measured on a Bruker ALPHA II spectrophotometer (Bruker, Billerica, MA, USA).

Unloaded Ce6 and siRNA were removed via centrifugation. The solution containing siRNA-Ce6 NPs was centrifuged at 10,000 rpm for 10 min; then, the free Ce6 and siRNA in the supernatant was determined by UV-Vis spectroscopy and the fluorescence spectrum, respectively. The encapsulation efficiency (EE) was calculated according to the following equation: [(amount of total Ce6 or siRNA − amount of Ce6 or siRNA in the supernatant)/amount of total Ce6 or siRNA] × 100%. The loading content was calculated by the following equation: (amount of Ce6 or siRNA in the precipitate/amount of the precipitate) × 100%.

### 3.4. Cell Culture

MCF-7 cells were cultured in MEM containing 10% FBS and 1% penicillin–streptomycin and maintained at 37 °C in a humidified atmosphere under 5% CO_2_.

### 3.5. Assessments of Cellular Uptake

The MCF-7 cell quantitative cellular uptake was analyzed using flow cytometry. MCF-7 cells were trypsinized and seeded in 6-well plates at a density of 1 × 10^5^ cells per well and incubated for 24 h. Then, the cells were treated with various concentrations of siRNA-Ce6 NPs (2, 4, 8 μg mL^−1^ of Ce6 and 0.06, 0.12, 0.24 μg mL^−1^ of siRNA) for specific incubation time periods. After incubation, the cells were trypsinized, collected by centrifugation, and washed with PBS. The cellular fluorescence intensity of ce6 and siRNA was assayed using flow cytometry (CytoFlex, Beckman Coulter, Pasadena, CA, USA). To conduct cellular bioimaging, MCF-7 cells were trypsinized and seeded in a 35 mm glass-bottomed dish. After 24 h incubation, the cells were treated with various concentrations of siRNA-Ce6 NPs (2, 4, 8 μg mL^−1^ of Ce6 and 0.06, 0.12, 0.24 μg mL^−1^ of siRNA) for specific incubation time periods. Finally, the cells were washed with PBS three times followed by fixation with 4% paraformaldehyde for 15 min. After staining with DAPI for 10 min, the cells were imaged by CLSM (ZEISS LSM 880, Oberkochen, Germany).

### 3.6. Cytotoxicity Evaluation

MCF-7 cells were seeded in 96-well plates at a density of 1.0 × 10^4^ cells per well and incubated for 24 h. Then, cells were treated with histone–HA NPs (0, 50, 100, 200, 500, and 1000 μg mL^−1^), Ce6 NPs (2, 4, 8 μg mL^−1^), siRNA NPs (0.06, 0.12, 0.24 μg mL^−1^), and siRNA-Ce6 NPs (2, 4, 8 μg mL^−1^ of Ce6 and 0.06, 0.12, 0.24 μg mL^−1^ of siRNA) for 24 h. Following this, cells were exposed to laser irradiation (at a wavelength of 660 nm and a power density of 0.2 W cm^−2^) for 1 min and continuously incubated for another 24 h. The viability of cells was then determined using the MTT assay. Additionally, the live–dead cell assay was performed to visualize the cytotoxicity of histone–HA NPs, Ce6 NPs, siRNA NPs, and siRNA-Ce6 NPs with and without laser irradiation. The cells underwent identical treatment before being stained with the Live/Dead Cell Staining Kit according to the manufacturer’s instructions. Finally, cells were imaged under CLSM.

### 3.7. Wound Healing Assay

MCF-7 cells were seeded in the well of a 6-well plate at a density of 1.0 × 10^4^ cells. After 24 h incubation, a line was scratched orthogonally using a sterile 200 µL micropipette tip and washed with PBS. Then, cells were treated with histone–HA NPs and siRNA-Ce6 NPs (2 μg mL^−1^ of siRNA) for 24 h, 48 h, and 72 h. Finally, the migration rates were calculated at each time point.

### 3.8. Measurement of Intracellular ROS

MCF-7 cells were seeded into 24-well plates (5 × 10^4^ cells per well) for 24 h. Subsequently, the cells were incubated with siRNA-Ce6 NPs (2, 4, and 8 μg mL^−1^ of Ce6) for an additional 24 h. Next, DCFH-DA (10 μM) was added and incubated with cells for 30 min. Afterwards, the cells were exposed to a 660 nm laser (0.2 W cm^−2^) for 1 min and soon imaged by fluorescence microscopy (U920FL, YUESCOPE, Hefei, China).

## 4. Conclusions

In summary, we successfully constructed histone-HA NPs by the electrostatic co-assembly strategy. Histone-HA NPs show high efficiencies in the co-loading of the photosensitizer Ce6 and HMGB1-siRNA. Particularly, histone-HA NPs can effectively deliver Ce6 and HMGB1-siRNA to cancerous cells to achieve robust PDT and gene editing, resulting in a significant breast cancer cell suppression effect. In addition, histone-HA nanoparticles exhibit good safety on cells due to their natural sources. Overall, co-assembled nanoparticles based on histone and HA provide a promising opportunity for the development of supramolecular drug delivery platforms toward combined gene therapy and PDT in cancer treatment. As gene therapy and PDT have wide applications in various pathologies [42,43], the co-assembled nanoparticles could be investigated for treating other diseases where the targeted delivery of siRNA and photosensitizers is beneficial, such as psoriasis- and age-related macular degeneration.

## Data Availability

The data that support the findings of this study are available in the figures and Supplementary Material of this article.

## Supplementary Materials

The following supporting information can be downloaded at https://www.mdpi.com/article/10.3390/ijms25105424/s1.
