# Supplementary material for: Supramolecular Nanoparticles of Histone and Hyaluronic Acid for Co-Delivery of siRNA and Photosensitizer In Vitro
Source: Int J Mol Sci. 2024 May 16;25(10):5424. doi: 10.3390/ijms25105424 (PMC11121309; doi:10.3390/ijms25105424)
Supplement: Supplementary file 1 [file ijms-25-05424-s001.zip › ijms-2989291-supplementary.pdf]

Supplementary Materials

Supramolecular Nanoparticles of Histone and Hyaluronic Acid for Co-delivery of siRNA and Photosensitizer *In Vitro*

Minxing Hu [1 †], Jianwei Bao [1 †], Yuanmei Zhang [1], Lele Wang [1], Ya Zhang [1], Jiaxin Zhang [1], Jihui Tang [1,*] and Qianli Zou [1,2,*]

[1] Research and Industrialization of New Drug Release Technology Joint Laboratory of Anhui Province, School of Pharmacy, Anhui Medical University, Hefei 230032, P. R. China

[2] Institute of Health and Medicine, Hefei Comprehensive National Science Center, Hefei 230000, P. R. China

[*] Correspondence: J. Tang, tangjh@ahmu.edu.cn; Q. Zou, qlzou@ahmu.edu.cn

[†] These authors contributed equally to this work.

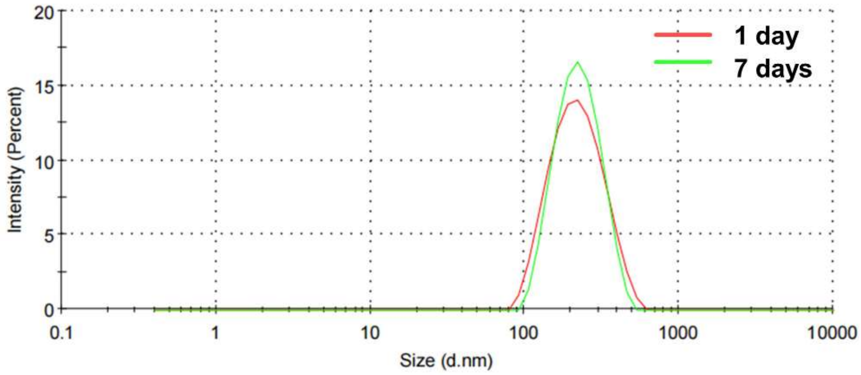

Figure S1. Size distribution of histone-HA NPs aged for 7 days.

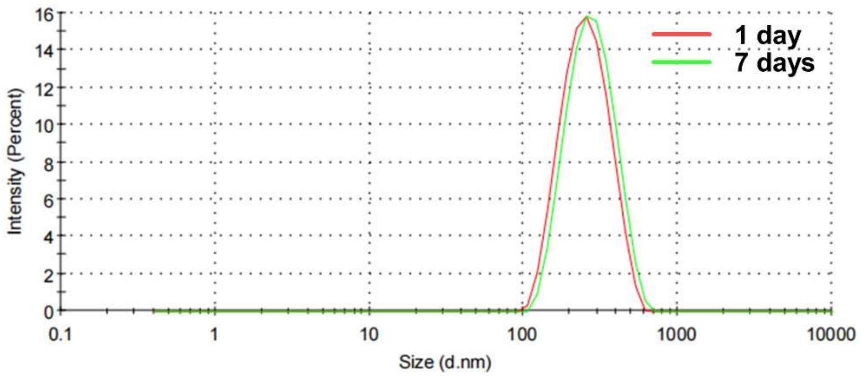

Figure S2. Size distribution of siRNA-Ce6 NPs aged for 7 days.

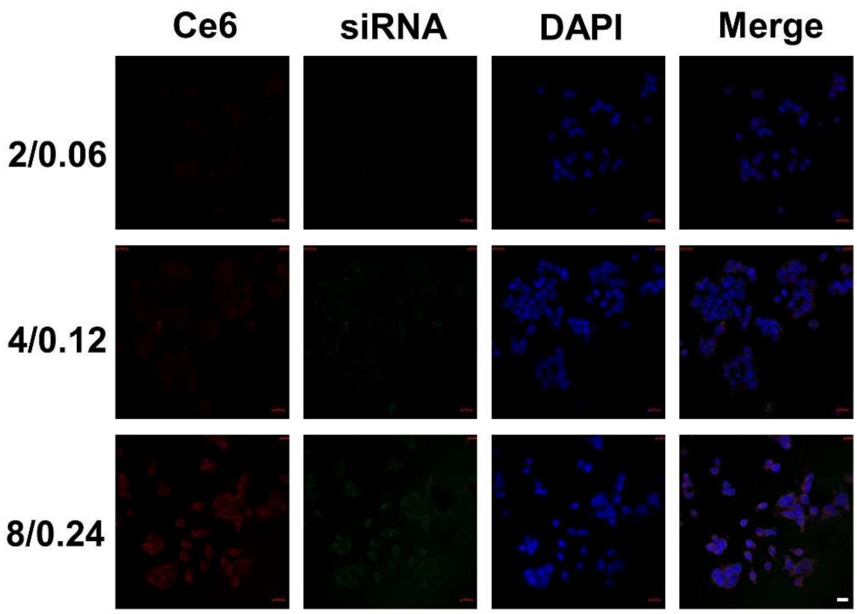

Figure S3. CLSM images of of MCF-7 cells incubated with siRNA-Ce6 NPs for 2 h. The concentrations of Ce6 in nanoparticles were 2, 4, and 8 μg mL^{-1}, respectively, and siRNA were 0.06, 0.12, 0.24 μg mL^{-1}, respectively. Scale bar: 20 μm.

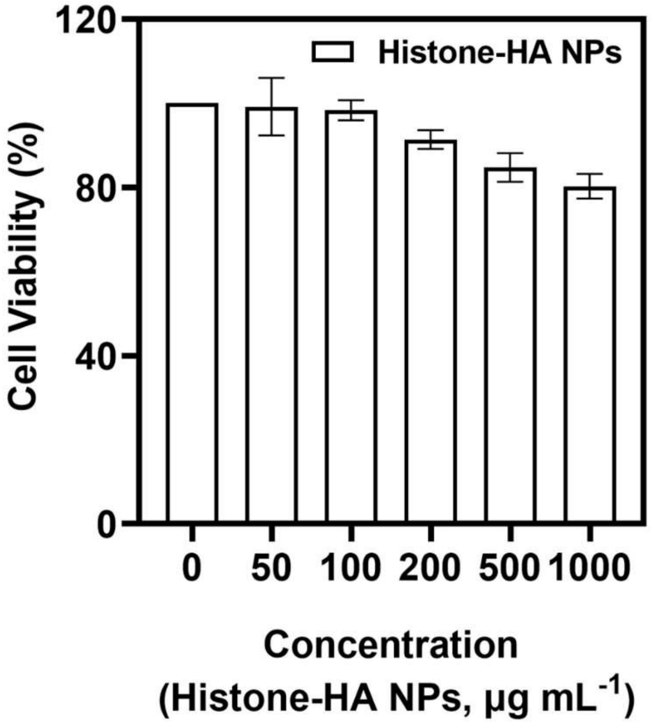

Figure S4. Cell viabilities of MCF-7 cells after treatment with Histone-HA NPs.

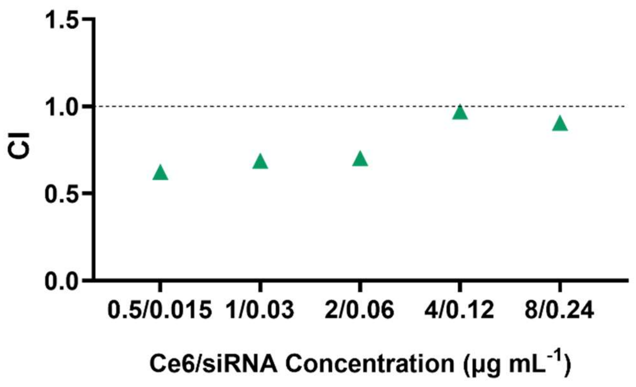

Figure S5. The combination index (CI) values under the different concentration of Ce6-siRNA NPs. CI < 1 is considered to be synergism.

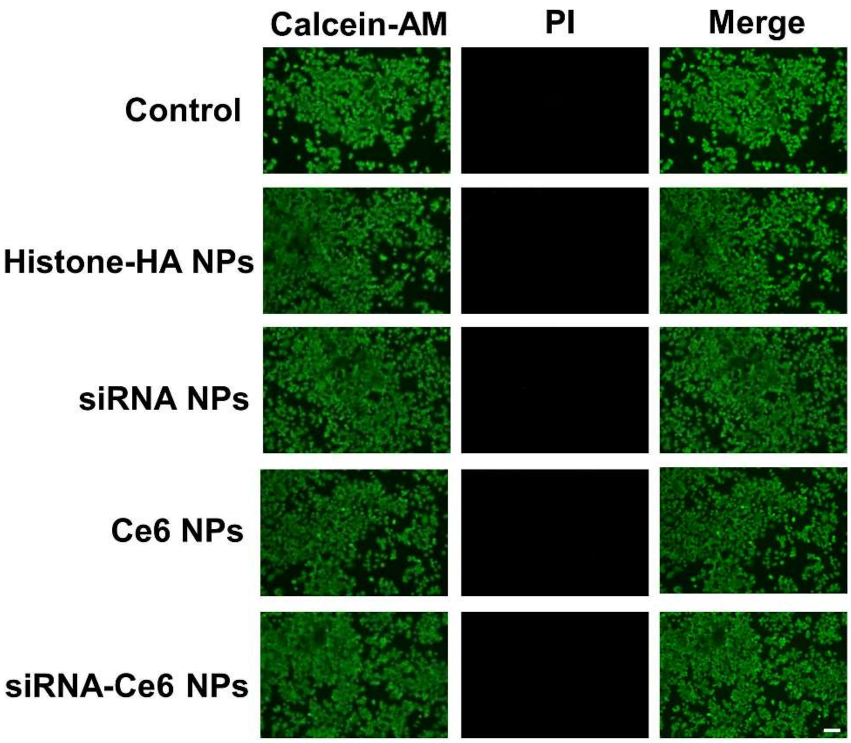

Figure S6. Calcein-AM and PI live-dead cell staining of MCF-7 cells after treatment with histone-HA NPs, siRNA NPs, Ce6 NPs, and siRNA-Ce6 NPs for 24 h. Scale bar: 50 μm.
